# Two Hernias, One Patient: A Case of Organoaxial Gastric Volvulus in a Paraesophageal Hernia With an Incisional Hernia

**DOI:** 10.7759/cureus.88523

**Published:** 2025-07-22

**Authors:** Ronit Biswas, Satendra Kumar, Sanjeev Gupta, Seema Khanna

**Affiliations:** 1 Department of General Surgery, Institute of Medical Sciences, Banaras Hindu University, Varanasi, IND

**Keywords:** advanced laparoscopy, incisional ventral hernia, laparoscopic incisional hernia repair, laparoscopic toupet fundoplication, organo-axial gastric volvulus

## Abstract

A 65-year-old male presented with abdominal pain and swelling persisting for five years. On clinical examination, a 15 cm transverse scar was noted above the umbilicus, along with a reducible incisional hernia. Contrast-enhanced CT of the abdomen revealed an incisional hernia with a single defect at the umbilical and epigastric regions with European Hernia Society classification of M2-3, W3, along with a rolling-type paraesophageal hernia. He underwent surgical repair for the incisional hernia by hybrid procedure. One month post-discharge, he developed progressive nausea, vomiting, and food intolerance. Repeat imaging and upper GI endoscopy suggested a large hiatal hernia with organoaxial gastric volvulus. Laparoscopic derotation with cruroplasty and Toupet fundoplication was performed with a successful outcome. This case illustrates a rare dual presentation of two distinct hernias, underscoring the need for comprehensive preoperative evaluation and discussing the possible factor of raised intra-abdominal pressure that can lead to gastric volvulus.

## Introduction

A hiatal hernia is a condition where part of the stomach or other intra-abdominal organs herniates through the esophageal hiatus of the diaphragm into the thoracic cavity. The gastroesophageal junction (GEJ) typically lies below the diaphragm; in hiatal hernia, its position may be altered, compromising the lower esophageal sphincter and predisposing individuals to gastroesophageal reflux disease, nausea, and vomiting.

The incidence of gastric volvulus in patients with hiatal hernia varies, but it is estimated to occur in approximately 5-28% of cases, particularly in those with paraesophageal or mixed-type hernias. The risk increases with large hernia size and laxity of the diaphragmatic hiatus [1].

There are four types of hiatal hernias: type I (sliding), where the GEJ moves above the diaphragm (95% of cases); type II (paraesophageal), where the fundus herniates beside the esophagus and the GEJ remains in place; type III, a combination of sliding and paraesophageal hernia; and type IV, herniation of the stomach along with other organs like the colon, small intestine, or spleen [2].

Hiatal hernias are more common in the elderly due to age-related degeneration of the diaphragm. Other predisposing factors are increased intra-abdominal pressure as a result of obesity, pregnancy, chronic obstructive pulmonary disease, chronic constipation, or any previous surgery [3].

## Case presentation

A 65-year-old male farmer presented to the surgery outpatient department (OPD) of a tertiary care center with a five-year history of abdominal pain and swelling, which increased on straining and coughing and was relieved by lying down and resting. On physical examination, a 4×3 cm swelling was noted in the supraumbilical region, soft and doughy in consistency, with ill-defined margins, manually reducible, and with a positive cough impulse. There was also a 6×5 cm swelling in the epigastric region, which was similarly soft, partially reducible, and had a positive cough impulse. A 15 cm transverse scar was noted above the umbilicus.

**Figure 1:**
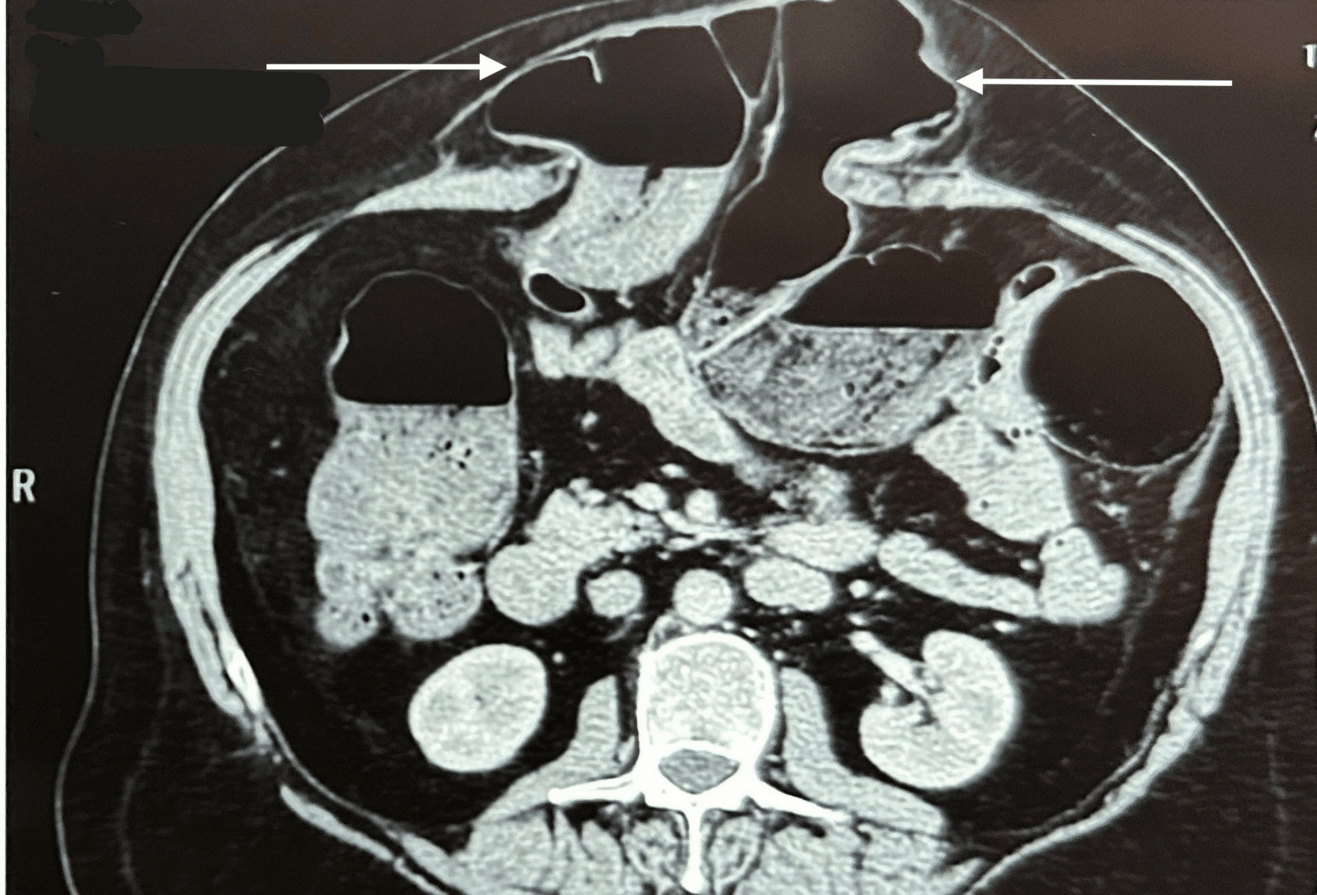
CECT showing previous incisional hernia with herniating large bowel, small bowel, and omentum in a large midline defect: M2-3, W3 CECT: contrast-enhanced computed tomography

The patient gave a history of abdominal surgery with mesh placement approximately 30 years ago at a local hospital, though no documentation was available. Contrast-enhanced CT (CECT) of the abdomen revealed a midline epigastric abdominal wall defect measuring 12 cm × 8.4 cm, containing omentum and a dilated loop of transverse colon, consistent with an epigastric incisional hernia (EHS: M2-3, W3) (Figure 1).

A type II (paraesophageal) hiatal hernia was also identified, with a significant esophageal hiatus defect and herniation of the stomach, while the GEJ remained in its normal position.

The patient was a known case of hypothyroidism and was receiving thyroxine 100 mcg daily. The patient was initially planned for surgical repair of an incisional hernia due to a significant and long-standing hernia. He underwent a hybrid approach (limited open suture repair with IPOM-plus technique). His postoperative course was uneventful, and he was discharged in a stable condition.

Approximately one month later, he began experiencing progressive nausea, retching, and intolerance to solid foods, which later progressed to liquids. He also reported infrequent bowel movements. With symptoms unrelieved by oral medications, he returned to the surgery OPD after two months and was admitted for further evaluation.

**Figure 2:**
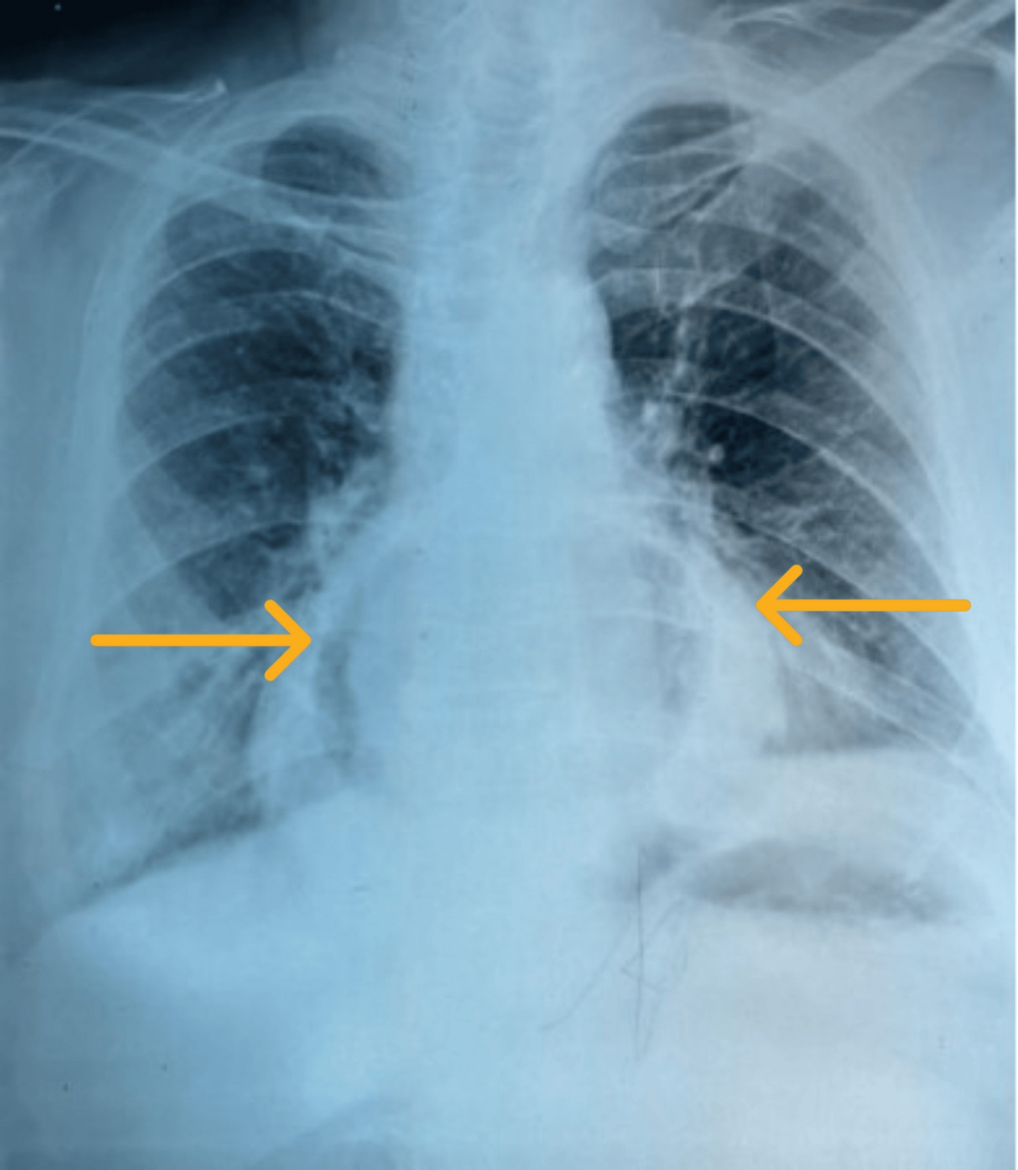
Chest X-ray showing thoracic gastric bubble

**Figure 3:**
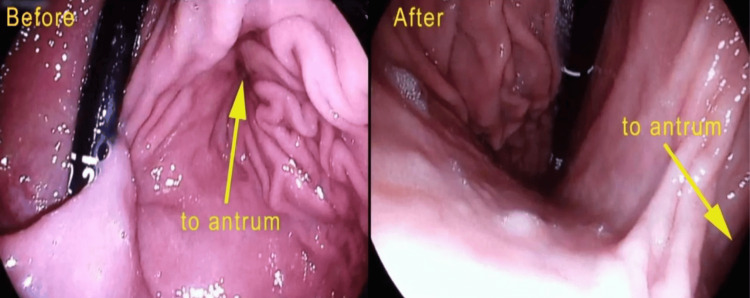
Paraesophageal hernia noted during esophagogastroduodenoscopy. The first image shows before retroflexion of the scope, and the second image shows after retroflexion of the scope

**Figure 4:**
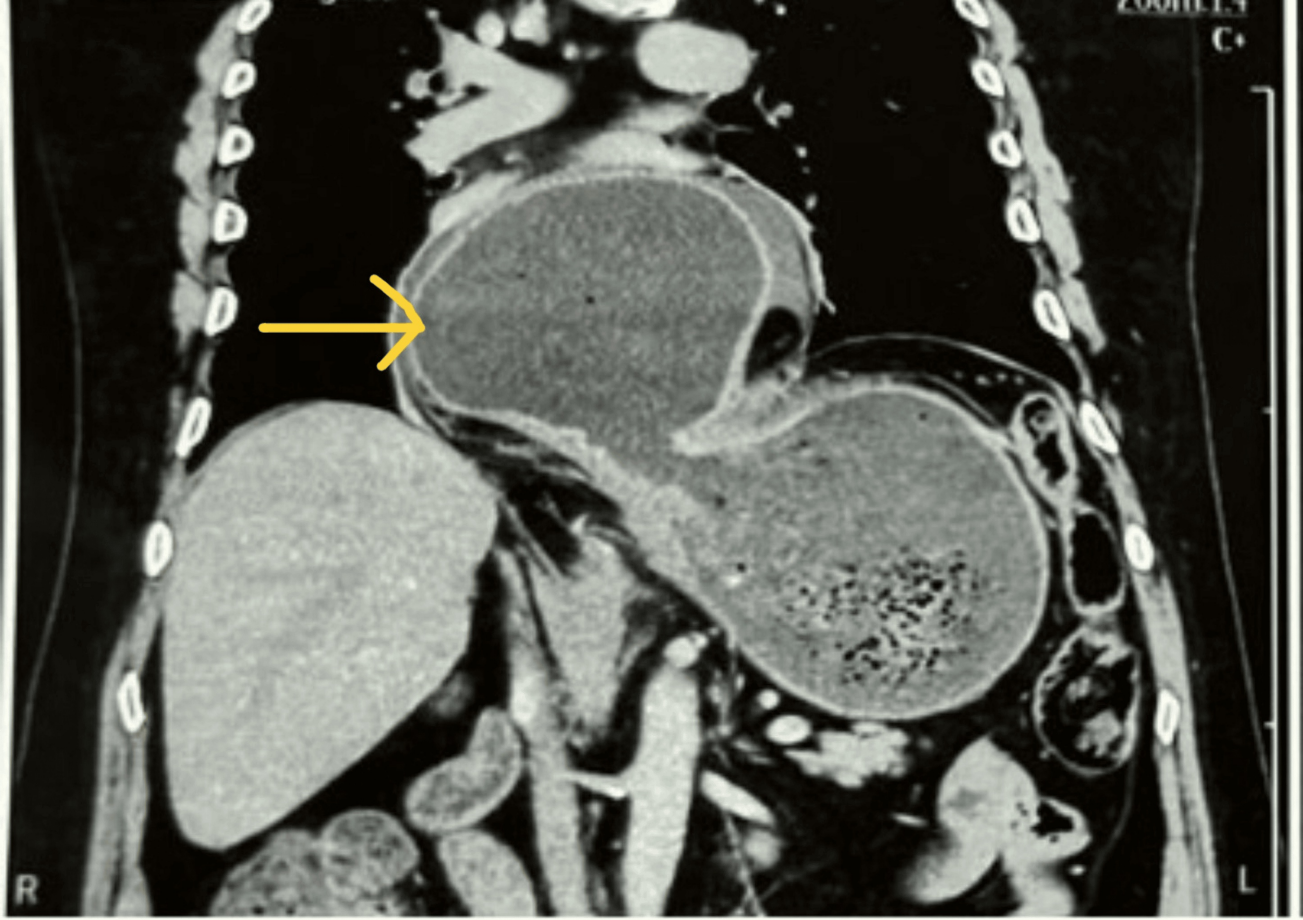
CECT showing herniated stomach in the thoracic cavity CECT: contrast-enhanced computed tomography

He was kept nil per os. A chest x-ray was done, which showed a gastric shadow in the mediastinum (Figure 2). Upper GI endoscopy revealed an inability to visualize the pylorus and a significant paraesophageal defect, with evidence of stasis esophagitis (Figure 3). A repeat CECT abdomen confirmed the presence of an organoaxial gastric volvulus associated with a hiatal hernia (Figure 4).

**Figure 5:**
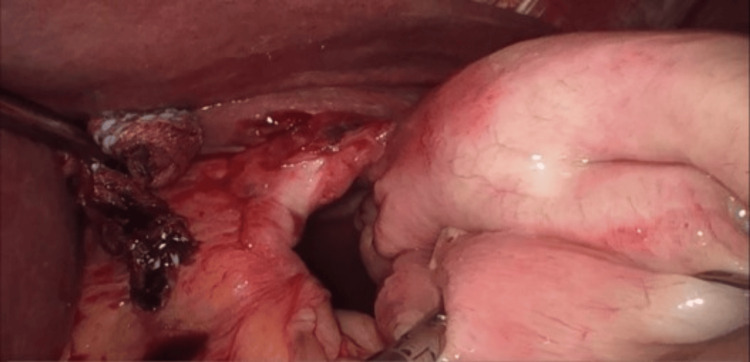
Intraoperative view showing hiatal hernia defect and viable stomach with no signs of strangulation

He underwent laparoscopic derotation of the volvulus and adhesiolysis between the hernial sac and stomach wall, and the hernial sac was reduced entirely from the thoracic cavity. After complete reduction, a wide hiatus was visible, for which cruroplasty was done with polyethylene 2-0 non-absorbable suture, and a partial wrap of 270° (Toupet’s fundoplication) was done to reduce postoperative bloating (Figure 5). The postoperative period was uneventful; the patient was allowed oral intake on postoperative day 3 after having an adequate bowel movement. Vigorous chest physiotherapy was recommended, along with a soft diet. The patient was discharged on postoperative day 5.

## Discussion

Gastric volvulus is a rare clinical entity characterized by an abnormal rotation of the stomach exceeding 180°, resulting in a closed-loop obstruction that may lead to incarceration and strangulation of the stomach, as stated by Milne et al. [4]. Akhtar et al. [5] have noted that gastric strangulation is a serious complication that can follow acute gastric volvulus, often progressing rapidly if not promptly diagnosed and treated. Early identification and timely surgical intervention are crucial to reducing associated morbidity and mortality.

Gastric volvulus may occur secondary to anatomical abnormalities such as diaphragmatic eventration, paraesophageal hiatal hernia, laxity or absence of the gastric ligaments, or postsurgical adhesion. It may also arise idiopathically, particularly in elderly patients with degenerative changes in their connective tissue.

Patients with gastric volvulus may present with a range of symptoms. Some exhibit only mild abdominal pain, nausea, and retching, while others present with sepsis due to gastric necrosis. In 1904, Borchardt described a classic triad for diagnosing acute gastric volvulus, consisting of (i) severe epigastric pain, (ii) retching without vomiting, and (iii) inability to pass a nasogastric tube.

The most widely accepted classification system for gastric volvulus, proposed by Singleton, is based on the axis of rotation and includes three typetypes: (i) organoaxial, rotation along the long axis connecting the cardia and pylorus; (ii) mesenteroaxial, rotation along the transverse axis from the lesser to greater curvature; and (iii) combined, features of both organoaxial and mesenteroaxial types [3,4].

In our case, the patient had epigastric pain along with retching with inability to vomit, and a nasogastric tube could not be passed successfully, thus fulfilling Borchardt's triad. However, in many cases, patients fail to fulfill the triad and may cause a significant dilemma in the diagnosis; hence, the surgeon should be adequately versed in all atypical presentations of gastric volvulus. Based on the CT findings, our patient had an organoaxial type of gastric volvulus. Management of acute gastric volvulus typically necessitates emergency surgical repair. For patients who are unfit for surgery, endoscopic reduction may serve as a temporary measure to relieve obstruction and allow for stabilization before undergoing definitive management. In chronic gastric volvulus, elective surgical intervention can be planned, often using laparoscopic techniques, which have shown favorable outcomes [6].

Clinically, gastric volvulus may present acutely, with severe epigastric pain, chest discomfort, and respiratory symptoms, or chronically, with intermittent epigastric pain, early satiety, and nausea. In some cases, it may mimic severe cardiac conditions like myocardial infarction due to overlapping symptoms. The choice between laparoscopic and open surgical approaches depends on the patient's clinical condition, urgency, and intraoperative findings. While acute cases demand urgent intervention, mainly when ischemia or gangrene is suspected, chronic cases in high-risk patients may be safely managed with less extensive procedures such as gastropexy (Table 1). The table below illustrates the variable nature of this condition's presentation. While abdominal pain was a constant feature in all mentioned case reports, chest pain was present in only two cases. The presenting state of patients is vital in deciding the outcome of the patient, as in case 1, the patient had come in emergency with unstable vitals and on endoscopy was found to have gastric necrosis, and the patient could not be saved. All other patients mentioned had a favorable outcome after operative management was performed, indicating that definitive management remains surgical correction.

**Table 1:** Comparison between other reported cases of gastric volvulus and their clinical presentation, management, and outcome

	Our case	Milne et al. [4]	Milne et al. [4]	Sachdeva et al. [7]	Akhtar et al. [5]	Longchamp et al. [8]	Qader and Abdul Hamid [6]
		Case 1	Case 2	Case 3	Case 4	Case 5	Case 6
Age	65 yr	46 yr	63 yr	85 yr	68 yr	72 yr	81 yr
Gender	Male	Female	Female	Female	Male	Female	Female
Pain abdomen	+	+	+	+	+	+	+
Chest pain	-	+	-	++	-	-	-
Vomiting	+	+	-	+	-	+	+
Presentation	Stable	Emergency	Stable	Shock	Stable	Stable	Stable
Type	Organoaxial	-	Organoaxial	-	Mesenterico-axial	Mesenterico-axial	-
Gastric necrosis	No	Yes	No	No	No	Yes	No
Management	Operative	Nonoperative	Operative	Operative	Operative	Operative	Operative
Outcome	Uneventful	Death	Uneventful	Uneventful	Uneventful	Uneventful	Uneventful

At the time of the initial presentation, the patient's primary complaints were related to the epigastric swelling, pain, and abdominal discomfort, which clinically and radiologically correlated with a large symptomatic incisional hernia. Although a paraesophageal hiatal hernia was identified on preoperative imaging, it was asymptomatic and not associated with significant gastroesophageal reflux or obstructive symptoms. Given the absence of acute or chronic symptoms attributable to the hiatal hernia at that time, and in light of the patient's age, comorbid condition (hypothyroidism), and the focus on resolving the symptomatic abdominal wall hernia, a staged surgical approach was adopted. It was decided to correct the hiatal hernia at a later date, and the patient was scheduled for a follow-up appointment. This decision aligns with clinical recommendations that advocate deferral of asymptomatic hiatal hernia repair to reduce operative complexity and surgical risk. The hiatal hernia became clinically significant only postoperatively, necessitating definitive surgical management when the patient developed features of gastric outlet obstruction due to organoaxial volvulus. This leads us to think whether high intra-abdominal pressure leads to the herniation and organoaxial gastric volvulus in this patient. Elevated intra-abdominal pressure can push the stomach upward, especially when support structures are compromised (e.g., hiatal hernia, eventration). This displacement increases gastric mobility and the risk of torsion. It can stretch and weaken the gastric ligaments, allowing for excessive sagging and twisting. Recent studies show that laparoscopic surgery yields good results for both acute and chronic gastric volvulus.

## Conclusions

Gastric volvulus is an uncommon and often unrecognized surgical emergency that should be suspected in patients who present to the hospital with severe epigastric pain, evidence of gastric outlet obstruction, and inability to pass the Ryle’s tube. Urgent laparotomy or laparoscopic management is needed to prevent serious complications like gangrene and perforation. This case report emphasizes the role of abdominal CT in diagnosing this life-threatening condition, making it the preferred diagnostic tool due to its availability. Upper GI endoscopy is also essential for diagnosis and ruling out complications, aiding in non-operative management for high-risk cases. Additionally, patients with incisional hernias and asymptomatic large hiatus hernias should undergo simultaneous laparoscopic repair to prevent severe complications, as seen in our case.
